# Practical considerations of linear accelerator‐based frameless extracranial radiosurgery for treatment of occipital neuralgia for nonsurgical candidates

**DOI:** 10.1002/acm2.12105

**Published:** 2017-05-18

**Authors:** Travis R. Denton, Lisa B.E. Shields, Jonathan N. Howe, Todd S. Shanks, Aaron C. Spalding

**Affiliations:** ^1^ The Norton Cancer Institute Radiation Center Norton Healthcare Louisville KY USA; ^2^ Associates in Medical Physics LLC Greenbelt MD USA; ^3^ Norton Neuroscience Institute Louisville KY USA; ^4^ The Brain Tumor Center Norton Healthcare Louisville KY USA

**Keywords:** extracranial SRS, frameless SRS, occipital neuralgia, quality assurance, stereotactic radiosurgery

## Abstract

Occipital neuralgia generally responds to medical or invasive procedures. Repeated invasive procedures generate increasing complications and are often contraindicated. Stereotactic radiosurgery (SRS) has not been reported as a treatment option largely due to the extracranial nature of the target as opposed to the similar, more established trigeminal neuralgia. A dedicated phantom study was conducted to determine the optimum imaging studies, fusion matrices, and treatment planning parameters to target the C2 dorsal root ganglion which forms the occipital nerve. The conditions created from the phantom were applied to a patient with medically and surgically refractory occipital neuralgia. A dose of 80 Gy in one fraction was prescribed to the C2 occipital dorsal root ganglion. The phantom study resulted in a treatment achieved with an average translational magnitude of correction of 1.35 mm with an acceptable tolerance of 0.5 mm and an average rotational magnitude of correction of 0.4° with an acceptable tolerance of 1.0°. For the patient, the spinal cord was 12.0 mm at its closest distance to the isocenter and received a maximum dose of 3.36 Gy, a dose to 0.35 cc of 1.84 Gy, and a dose to 1.2 cc of 0.79 Gy. The brain maximum dose was 2.20 Gy. Treatment time was 59 min for 18, 323 MUs. Imaging was performed prior to each arc delivery resulting in 21 imaging sessions. The average deviation magnitude requiring a positional or rotational correction was 0.96 ± 0.25 mm, 0.8 ± 0.41°, whereas the average deviation magnitude deemed within tolerance was 0.41 ± 0.12 mm, 0.57 ± 0.28°. Dedicated quality assurance of the treatment planning and delivery is necessary for safe and accurate SRS to the cervical spine dorsal root ganglion. With additional prospective study, linear accelerator‐based frameless radiosurgery can provide an accurate, noninvasive alternative for treating occipital neuralgia where an invasive procedure is contraindicated.

**Abbreviations**
CBCT: cone beam CT
DRR: digitally reconstructed radiograph
IEC: international electrotechnical commission
IGRT: image‐guided radiotherapy
OAR: organ‐at‐risk
PTV: planning target volume
SRS: stereotactic radiosurgery

## 1 INTRODUCTION

Occipital neuralgia is a neurological condition characterized by paroxysms of intense pain transmitted by the greater occipital nerves in the back of the head and neck often accompanied by a dull ache.^1^, ^2^, ^3^, ^4^ The condition is differentially diagnosed from other headache types by using patient descriptions of the pain, noting the location of tenderness associated with pain episodes, and achieving prompt relief of pain following an anesthetic block of the greater occipital nerve.^5^, ^6^ The incidence of occipital neuralgia in the general population remains unknown but is thought to be less than that of trigeminal neuralgia and glossopharyngeal neuralgia which have an incidence of 20/100,000 per year and 0.7/100,000 per year, respectively.^7^

Medical management for patients diagnosed with occipital neuralgia generally includes analgesics or anti‐inflammatories which proves effective for most patients. Numerous treatment options may be warranted for patients with continued disabling and intractable pain despite temporary treatments or when invasive therapies such as surgical incision, radiofrequency ablation, injected neurotoxin facilitated nerve blocking, implanted nerve stimulator, or surgically decompressing the nerve fail to provide relief.^6^, ^8^, ^9^, ^10^ If the condition continues to be refractory, nerve sparing procedures are utilized in preference to neurodestructive surgeries.^11^, ^12^, ^13^, ^14^ Neurodestructive procedures, such as neurectomies, are highly invasive and carry some degree of risk of permanent complication.^15^, ^16^ For those patients whose pain recurs following an invasive procedure, a secondary invasive procedure is often contraindicated due to compounding risk.^6^

Although occipital neuralgia has been reported to be a complication resulting from frame installation for frame‐based radiosurgery, the use of SRS for the treatment of occipital neuralgia has not been reported to date.^17^ The success of radiosurgery in the management of trigeminal and glossopharyngeal neuralgia (both cranial‐based functional diseases) has been well established.^18^, ^19^, ^20^, ^21^, ^22^, ^23^, ^24^, ^25^, ^26^ SRS for trigeminal neuralgia and glossopharyngeal neuralgia involves single‐fraction high doses to the isocenter placed along the course of the nerve after exiting the central nervous system. In comparison, radiosurgery for occipital neuralgia has not been well explored due to limitations in the ability of traditional SRS treatment modalities in delivering extracranial applications.^27^, ^28^

With the recent advances in intrafractional image guidance, we report here the treatment of occipital neuralgia using linear accelerator‐based frameless radiosurgery.

## 2 METHODS

### 2.A Phantom feasibility study

Quality assurance for geometric accuracy, precision, and dosimetric accuracy was established during commissioning of the functional SRS program. The initial commissioning included evaluation of cone positioning reproducibility, providing accuracy within 0.5 mm, and intercomparison of PDD, off‐axis factors, and total scatter factors with another institution. Principles of small‐field dosimetry were applied by measuring output with film, diode, and a small volume ionization chamber.^29^, ^30^, ^31^, ^32^, ^33^, ^34^ Relative output factors were obtained from the ratio of the dose at isocenter at depth *d*_*max*_ for the conical collimator (4 mm in our case) relative via the “daisy chain” method described by Dieterich et al. to the dose measured for a 100 by 100 mm^2^ square field size at a depth of *d*_*max*_ both at 1000 mm source to isocenter distance providing a ratio of 0.6668.^35^, ^36^ Total scatter factors compared against four other institutions results in agreement within 1%. The SRS single‐beam phantom from the Imaging and Radiation Oncology Core — Houston (IROC) was used to verify the output of the SRS cone program. The measurements and commissioning process received peer review by a medical physicist expert in radiosurgery as part of Novalis Certification.

A phantom feasibility study was performed by CT simulating, planning, and treating an SRS anthropomorphic head phantom (CIRS Computerized Imaging Reference System Inc. VA, USA). This phantom included simulated bony anatomy that is visible to both CT and x ray and included a neck which was necessary in consideration of the target localization under investigation.

**Figure 1:**
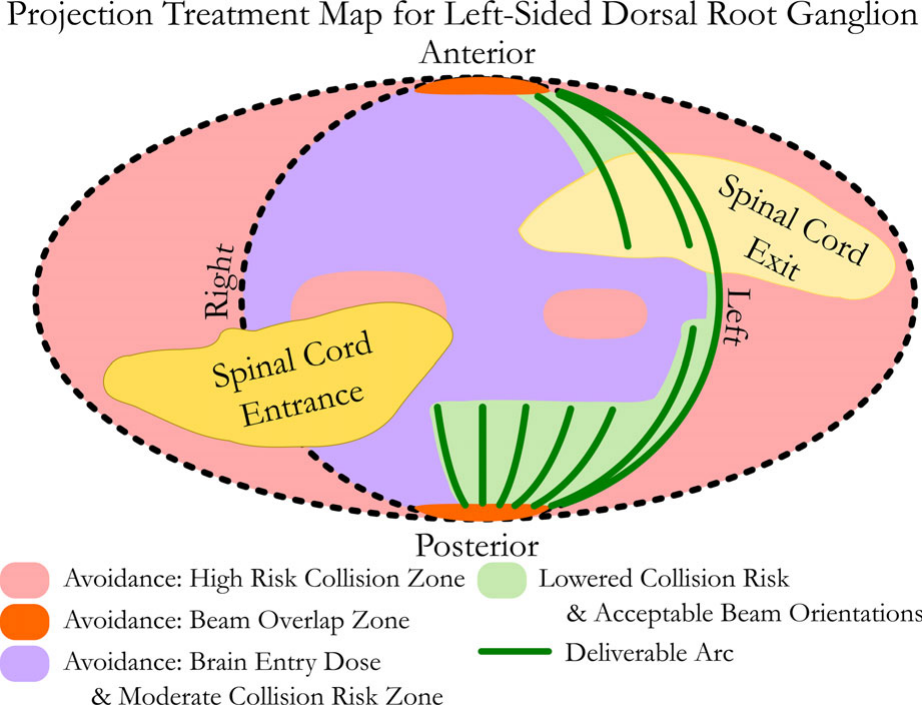
A projection treatment map for a left‐sided occipital neuralgia typical target is presented as a planning guide and result of a phantom feasibility test performed prior to attempting patient treatment. It is noted that a right‐sided target would need a guide that is mirror imaged and symmetric about the sagittal axis.

Figure 1 shows a treatment projection map established as a “planning guide” for typical patients. For interpretation purposes, this figure can be considered to be an elliptical projection to two‐dimensions of the three‐dimensional spherical space surrounding the patient's head with the patient in the treatment position. The central circle of Fig. 1 can be interpreted as the hemisphere of that spherical space corresponding to the superior hemisphere surrounding the patient's head. For additional orientation, consider the anterior pole to colloquially correspond to the patient's nose. It is not intended to be a “planning solution” for all patients whose anatomy could vary significantly. Patient‐specific collision avoidance verification tests were performed for the phantom and patient included in this study. Entry dose through the spinal cord was avoided by limiting or avoiding the use of beams entering through the contralateral side. The anterior and posterior poles were labeled as avoidance zones to prevent beam overlap. Brain entry dose was noted to avoid beams that would result in the beam first traveling through the brain to reach the target. Two elliptical shapes are represented on the left and right side of Fig. 1 that reflect two additional zones of high collision risk and represent the corners of the treatment couch used for this study which has rounded square corners and is not semicircular. Because of this design, there was an increased risk of collision as the conical collimator would start to dip below the horizon defined by the treatment couch with the contralateral side representing a larger risk area due to the lateral shift required to align the patient's target side to the radiation isocenter. The green zone represented a lowered collision risk. Due to this supine patient setup, radiation beams passed through the treatment couch prior to the patient. For this reason, the treatment couch and resultant attenuation was taken into account in the treatment planning system.

**Figure 2:**
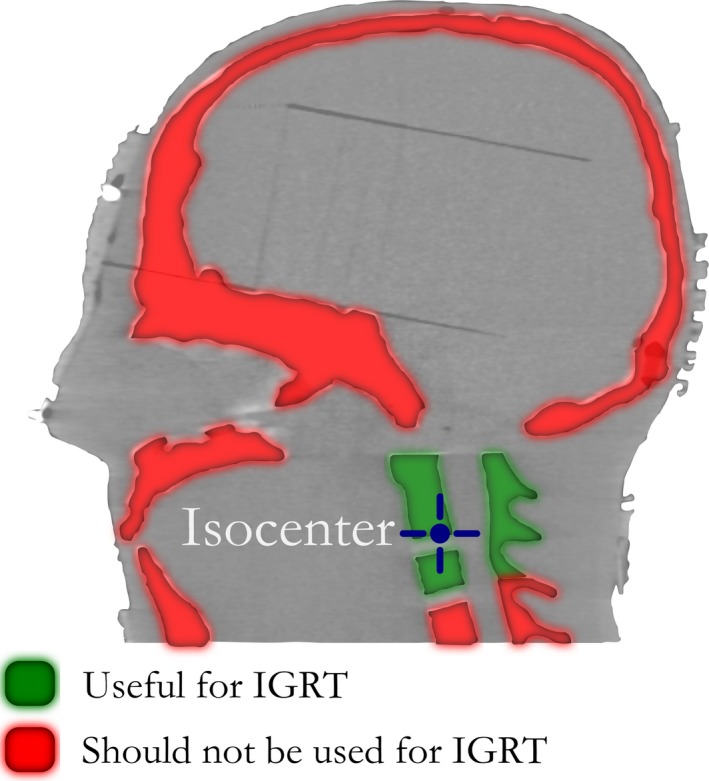
Image guidance for intrafractional positional corrections during the treatment of occipital neuralgia using a frameless approach requires an inverse approach as compared to traditional cranial‐based SRS. Image registration for IGRT during treatment should be performed using anatomy in the vicinity of the target (i.e., on the C1/C2 level). Other bony anatomy such as the skull, mandible, and the lower C‐spine should be understood as capable of moving independent of the target and, therefore, should not be used as registration references when aligning the patient to the isocenter.

Figure 2 was established to identify a meaningful image registration strategy as this target represented a deviation from the normal cranial‐based applications typically seen for the SRS workflow. Furthermore, only a small volume of the relevant anatomy would be useful for IGRT while a large volume of anatomy could be construed as confounding (including both fixed and mobile cranial anatomy). The figure presented here is composed as a simple sagittal illustration so that these points can be emphasized as failure to consider the nonstandard implications of these conclusions could result in coregistration performed to nonrelevant anatomy and possible target miss. The image registration accuracy of both planning and treatment image sets was vital to the successful delivery of this treatment due to the small target size, small beam aperture, and thin imaging slices.

### 2.B Case selection

For this case study, a 53‐year‐old male patient presented with complaints of severe cervical neck pain diagnosed as occipital neuralgia. The patient had undergone both radiofrequency ablation with 1 month of relief and cervical spine decompression and fusion of C4‐7 with no improvement. Various local injections provided only short lasting relief. The pain was reported to be debilitating with the maximum score of V on the Barrow Neurological Institute pain intensity scale.^37^ As the occipital neuralgia condition was refractory to radiofrequency ablation, surgery, and medical management, the option of radiosurgery was presented.

### 2.C Anatomy, targeting, and immobilization

An extended frameless SRS mask with eight fixation points was fabricated (frameless SRS mask set extended, BrainLab). This immobilization mask extended to include the patient's shoulders, as opposed to the standard cranial SRS mask which only immobilizes the head. This mask consisted of four fixation points on both sides of the patient with one at eye level, one at chin level, one at shoulder level, and one at the level of the armpit. To help ensure a higher degree of reproducibility of neck flexion from simulation to the time of treatment, a Moldcare^®^ cushion (Model: RT‐4492, QFix, Avondale, PA, USA) designed for head and neck immobilization was embedded in the mask setup. This custom cushion, as opposed to standard patient nonspecific head rests, improved immobilization and reproducibility, mainly through comfort by providing a resting surface for the cervical spine. The CT simulation was 0.6 mm in slice thickness and was completed with a localization box in a standard position to facilitate spatial localization of the CT dataset within the treatment planning system.

**Figure 3:**
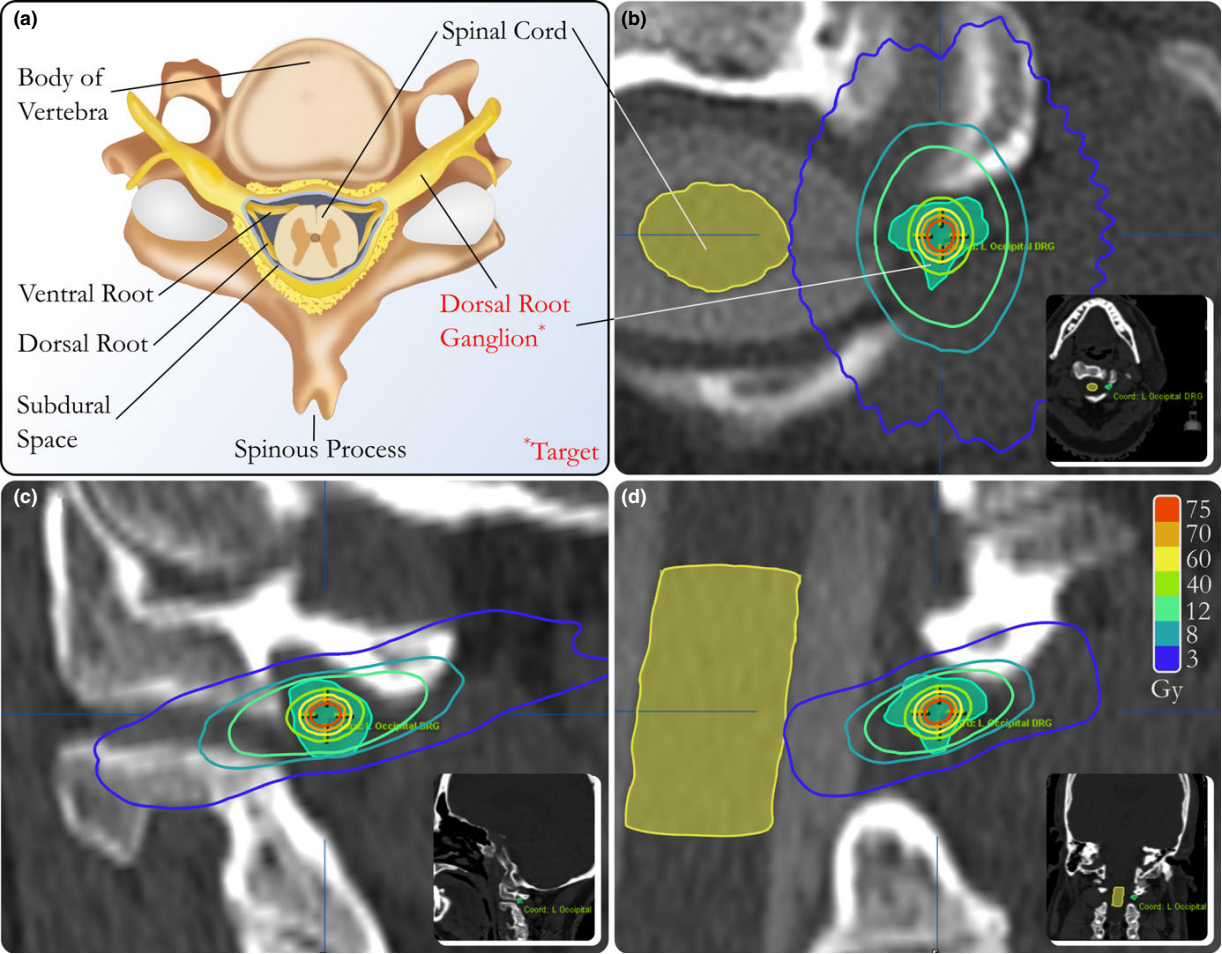
This illustration (a) and corresponding CT views (b, c, and d) demonstrate the target for occipital neuralgia as the center of a neurosurgeon contoured dorsal root ganglion of the occipital nerve corresponding to the lateral side of the patient's pain manifestation. Isodose lines generated by the treatment planning system correspond to a treatment plan generated for this target.

**Figure 4:**
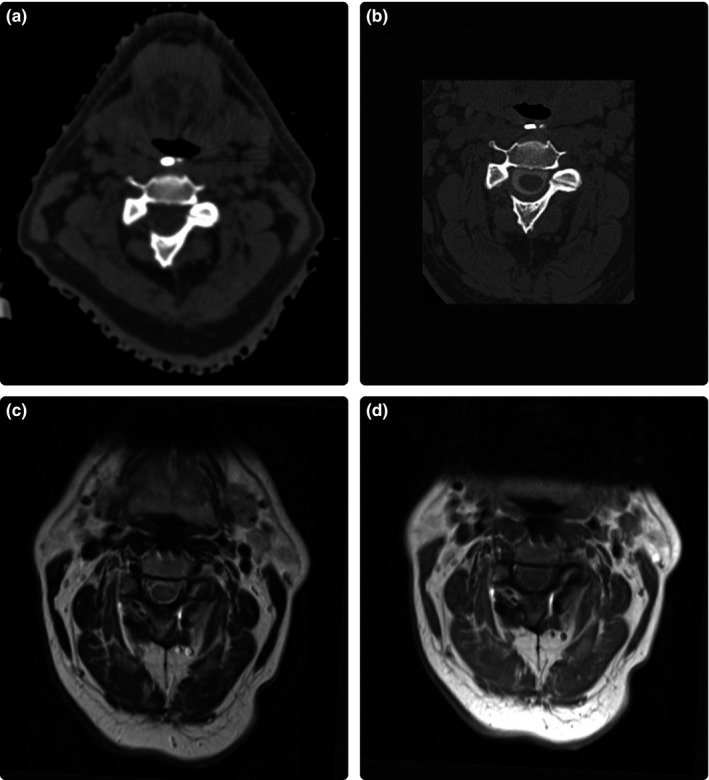
(a) CT simulation acquired at 0.6 mm slice thickness. Used as a reference set for dose calculations in the treatment planning system and localized using equipment and procedures corresponding to the ExacTrac workflow. (b) CT myelogram acquired at 1.0 mm slice thickness. (c) T2‐weighted MRI acquired at 3.0 mm slice thickness. (d) T1‐weighted MRI acquired at 3.0 mm slice thickness.

The therapeutic target was the dorsal root ganglion of the occipital nerve on the side associated with the pain. A thin‐slice (1.0 mm) CT myelogram was necessary to adequately visualize the target. The coregistration used to align the target definition imaging to CT simulation was performed by the medical physicist and then reviewed with the medical physicist, radiation oncologist, and neurosurgeon. A local rigid transformation to the CT myelogram was performed with a volume of interest (VOI) centered on the spinal cord at the C2 level. The rectangular VOI was selected to include the entirety of the C2 vertebrae and portions of C1 and C3. This resulted in a VOI with dimensions of: 62.7 mm left‐to‐right, 54.9 mm anterior‐to‐posterior, and 46.4 mm superior‐to‐inferior. The dorsal root ganglion was contoured by the neurosurgeon, and the isocenter for the radiation treatment plan was aligned to the center of this contour. Figure 3 shows an anatomical illustration of the intended target along with axial, sagittal, and coronal views of the target. The CT myelogram was preferable for target definition, in this case, to MRI for two reasons. First, artifacts from the titanium hardware were present on MRI. Secondly, like‐modality image registration was preferable to cross‐modality image registration in an effort to decrease uncertainty introduced in the image registration step.^38^ Figure 4 shows all available image modalities.

X‐ray image guidance was performed throughout the treatment delivery, and the results of the ExacTrac‐based coregistration algorithm were carefully evaluated with each treatment couch angle and prior to the delivery of each treatment beam. Imaging was obtained as necessary based on infrared marker monitoring and/or suspected patient movement. The radiation oncologist remained throughout the treatment delivery and provided immediate physician review of the intrafractional imaging. Furthermore, the referring neurosurgeon, coresponsible for delineating the treatment target during planning, was present during treatment delivery to lend expertise to the analysis of the IGRT.

### 2.D Treatment and IGRT equipment

The treatment was successfully performed with a NovalisTX (Varian Medical Systems, Palo Alto, CA, USA) equipped with ExacTrac stereoscopic x‐ray image guidance (BrainLab AG, Feldkirchen, Germany). The treatment couch was an IGRT couch top (BrainLab AG, Feldkirchen, Germany). We previously determined the congruency between mechanical (including gantry and couch rotational isocenters), IGRT, in‐room laser, and treatment beam isocenters specific for this radiation treatment device and imaging platform.^39^ This study quantified an average magnitude of distance for the laser‐defined alignment isocenter to be 0.58 mm. The effect of gantry sag was 0.4 mm in magnitude. The IGRT isocenter was within 0.5 mm of the radiation‐defined isocenter. Couch walkout had a maximum discordance of 0.72 mm with the isocenter. These values were based on a statistical analysis of 149 individual isocenter congruency tests performed for this machine and imaging combination. The magnitude of difference between the radiation‐defined and ExacTrac IGRT‐defined isocenter was significantly less than the couch walkout. Therefore, imaging was performed for each treatment couch angle prior to treatment arc delivery.

## 3 RESULTS

### 3.A Phantom feasibility study

A projection treatment map was established following the CT simulation of the anthropomorphic head phantom with a physician‐provided isocenter and treatment target corresponding to that which would be spatially and characteristically used for occipital neuralgia. This map reflected a treatment strategy that would ensure the following: (a) clearance could be achieved without collision for all treatment angles and with the ancillary treatment cone mounted, (b) a sufficient number of arc angles could be achieved from a suitable number of unique angles that would make this high dose stereotactically achievable with quality gradient indices, and (c) normal tissues such as the brain and entry dose through the spinal cord could be achieved without comprising the desired treatment dose. This projection map is shown in Fig. 1 and is intended as a planning guide for the treatment of frameless linear accelerator‐based SRS for occipital neuralgia. It was concluded that a typical occipital neuralgia treatment plan could be delivered using approximately 540 arc degrees while avoiding collisions, unnecessary brain dose, and entry dose through the spinal cord.

After establishing the parameters for simulation, fusion, and treatment planning, the same phantom was used to define the conditions for treatment delivery with BrainLab's ExacTrac image guidance system. Infrared markers were used to provide the initial setup and x rays were acquired to match to bony anatomy. A simulated treatment was performed with additional intrafractional imaging performed at each treatment couch position. The average translational magnitude of correction to realign the phantom's vertical, longitudinal, and lateral position was 1.35 mm with an asserted acceptable tolerance of 0.5 mm or less as not requiring a correction. The average rotational magnitude of correction applied to correct the phantom's pitch, yaw, and roll was 0.4° with an acceptable tolerance for each couch position of 1.0° or less not requiring a correction. The IGRT guide provided in Fig. 2 was consistent with our expectations with the bony anatomy useful for IGRT providing a sufficient amount of image information for accurate image registration during a treatment situation.

### 3.B Plan characteristics

The dose grid resolution in the treatment planning system was set to 0.5 mm. For small objects, such as the contoured target, the grid size was automatically adjusted such that at least 10 voxels for each dimension were included inside the small planning target volume (PTV) or organs‐at‐risk (OAR) used in the dose algorithm calculation. At its closest point, the spinal cord was measured to be 12.0 mm from the isocenter. Eighty gray in a single fraction was delivered to the isocenter using 9 mostly ipsilateral 4 mm cone‐based arcs with 6 MV photons. The spinal cord received: D(1.0 cc) = 0.97 Gy, D(0.1 cc) = 2.55 Gy, and a maximum dose of 3.36 Gy. The brain received: D(10.0 cc) = 1.19 Gy and a maximum dose of 2.20 Gy.

A gradient index was evaluated for this plan based on a metric established for a cohort of functional disease patients treated using this linear accelerator and conical collimated SRS beam. The plans in this cohort were taken from plans approved for treatment by the radiation oncologist from the established frameless functional disease SRS program (trigeminal neuralgia, *n* = 78, glossopharyngeal neuralgia, *n* = 1). The gradient index for all of the plans (*n* = 80) was calculated using the definition from Paddick et al. as the ratio of the 25% isodose volume and the 50% isodose volume.^40^ These values were chosen because the nature of these plans resulted in a point dose to the 100% prescription dose placed at the geometric center of the nerve to be treated. The gradient index from our metric cohort was 3.431 ± 1.067 which was compared to our case study gradient index value of 3.494.

Postplan analysis was performed by developing a composite plan with each arc's isocenter modified from the shared planning isocenter by that arc's residual spatial corrections as identified by IGRT and below the threshold required for positional correction determined immediately prior to the arc's delivery. Postplan dosimetry determined that the spinal cord received the following: D(1.0 cc)= 0.96 ± 0.02 Gy, D(0.1 cc) = 2.54 ± 0.06 Gy, and a maximum dose of 3.59 ± 0.08 Gy. Furthermore, the brain received: D(10.0 cc) = 1.19 ± 0.03 Gy and a maximum dose of 2.22 ± 0.05 Gy.

### 3.C Image guidance

**Figure 5:**
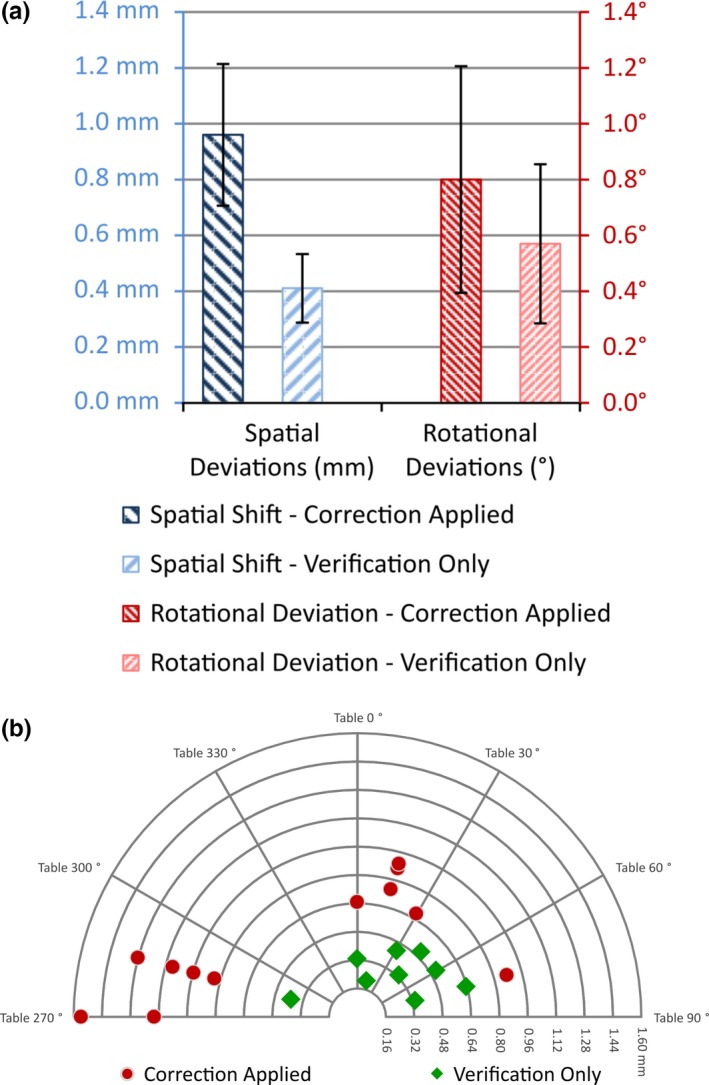
(a) The average magnitude of deviation for patient requiring a positional correction was 0.96 ± 0.25 mm/0.8 ± 0.41°, whereas the average magnitude of shift considered to be within tolerance was 0.41 ± 0.12 mm/0.57 ± 0.28°. The maximum deviations were 1.55 mm/1.1°. This plot illustrates the global degree of intrafractional motion differentiated by whether or not a positional/rotational correction was deemed necessary. (b) Magnitudes of spatial deviations for patient as a function of couch position for all intrafractional image guidance. Couch positions were captured via an array of infrared markers and calibrated camera system as a function of the image guidance system (IEC accelerator convention).

The results of intrafractional IGRT are graphically represented in Figs. 5(a) and 5(b). Linear accelerator equipment parameters are presented using the International Electrotechnical Commission (IEC) accelerator convention.^41^ The treatment time was 59 min for 18,323 MUs with imaging being performed at each treatment couch position, i.e., prior to each arc delivery for a total of 21 stereotactic image pairs for intrafractional alignment verification. Twelve of these required repositioning and subsequent reimaging for verification. The average deviation magnitude requiring a positional or rotational correction was 0.96 ± 0.25 mm, 0.8 ± 0.41°, whereas the average deviation magnitude deemed within tolerance was 0.41 ± 0.12 mm, 0.57 ± 0.28°. Spatial deviations, once corrected, are significantly improved using error analysis with an accepted threshold of 0.5 mm. Rotational deviations, on the other hand do not show a significant improvement postcorrection with an accepted threshold of 1.0°. For this treatment, more stringent tolerances were applied for spatial versus rotational corrections because a spatial target miss would not be acceptable, whereas rotational deviations would not change target dose due to the large number of arc angles employed and the isotropic nature of the dose distribution.

### 3.D Patient outcome

At most recent follow‐up, the patient reported 4 months of pain relief. No subsequent radiation was delivered. The patient is currently being followed by neurosurgery. No repeat imaging has been done to date.

## 4 DISCUSSION

SRS for functional diseases including neuralgias has been well established with demonstrable precision using both frame and frameless approaches.^18^, ^19^, ^20^, ^21^, ^22^, ^23^, ^24^, ^25^, ^26^ Kim et al. demonstrated that the NovalisTX equipped with ExacTrac was capable of approximately 1 mm accuracy for localizing targets.^42^ Advances in image‐guided systems and frameless SRS capabilities have allowed for the consideration of new treatment sites that have been previously restricted due to the limitations of historic SRS modalities. While conditions such as trigeminal and glossopharyngeal neuralgia have been successfully treated using SRS, occipital neuralgia treated with any type of SRS modality and immobilization technique has not, to date, been reported in literature. Demonstration of this successfully delivered SRS dose using a frameless SRS approach provides an important alternative for patients suffering from occipital neuralgia and who have exhausted traditional pain management strategies.

Radiosurgery has evolved from frame‐based approaches to frameless over recent years, providing advantages in clinical workflow and intrafraction patient monitoring.^43^, ^44^, ^45^ More recently, stereotactic doses have been successfully applied to extracranial applications which are largely frameless.^46^, ^47^, ^48^, ^49^ The treatment of extracranial functional disease using radiosurgery has not been previously reported with either frame‐based or frameless systems. The use of stereotactic cones on gantry mounted linear accelerator presents an increased risk of collision that is well understood for cranial applications but requires more detailed review for extracranial applications. Furthermore, the use of a stereotactic frame may preclude treatment for occipital neuralgia. Most frames are designed with the frame‐to‐table mount positioned inferior to the base of skull, at the same level as the occipital nerve. The frame would present challenges to treatment planning as beams would pass through the frame. Recent advances in Gamma Knife technology allow for SRS treatments to be delivered extracranially for the cervical spine and head and neck applications. CyberKnife offers the capability of being able to treat at this treatment level. However, no application of these treatment modalities have yet to be reported in the literature.

This study demonstrated that a cone‐based stereotactic treatment was devisable and deliverable with a sufficient number of treatment arc degrees while mitigating table, immobilization device, and patient collision risk. Limitations imposed by avoiding unnecessary beam entry dose through the brain, beam overlap, and beam entry through the spinal cord were identified and overcome. Secondly, this study was used to verify the feasibility that accurate image‐guided radiotherapy (IGRT) could be achieved for this treatment site in consideration that conventional image guidance using fixed bony cranial anatomy would not provide relevant image registration anchors for this treatment target. The phantom and, subsequently, the patient underwent a supine setup. There may be additional value in considering prone‐based setups. However, for the image guidance system used, ExacTrac prone positioning is not supported.

End‐to‐end testing using this modality was completed during the commissioning of both the linear accelerator and beam model in the treatment planning system used for planning in this study in accordance with current standards of practice.^35^, ^50^, ^51^, ^52^, ^53^, ^54^, ^55^, ^56^, ^57^, ^58^, ^59^, ^60^ In addition, a separate study performed for this treatment modality characterized the submillimeter congruence of isocentricity of the radiation, mechanical, and image guidance components used for this case study.^39^ Due to the target size and location, any linear accelerator attempting to perform SRS for occipital neuralgia would need to undergo specific end‐to‐end testing with a phantom before treating a patient. Furthermore, during our analysis, couch walkout contributed to a significant degree of incongruence to the radiation‐defined isocenter. Therefore, pretreatment x‐ray imaging and patient position correction were performed for each treatment couch angle. Other linear accelerators may have different factors requiring other methods of correction. A study by Shanks et al. detailed the establishment of a program for frameless SRS treatment of functional disease with notable emphasis on the level of collaboration achieved between neurosurgery and radiation oncology which was instrumental in ensuring accurate target definition.^61^ A description of the commissioning efforts of the functional SRS program is outside of the scope of this case study. Rather, this treatment site is a new application for a mature functional disease SRS program. Commissioning included independent dose measurement audits provided by the IROC for both routinely measured absolute dose verifications and commissioning SRS‐specific phantom measurements performed prior to the clinical use of the treatment planning system for such applications. The results of these studies were within satisfactory limits as per IROC analysis.^62^ In addition, our clinic has participated and received accreditation from the American College of Radiology and is a Novalis Certified Radiosurgery Center.^63^, ^64^

From the treatment projection map, we determined collision avoidance verification tests are recommended for each patient. This facilitates keeping arcs ipsilateral to the treatment side and guided from the medial, anterior, and posterior aspects to mitigate collision risk and unnecessary brain dose. This technique resulted in isodose lines with elongated low‐dose regions in both the patient left‐to‐right and posterior‐to‐anterior directions. The maximum point dose was well situated in the center of the contoured dorsal root ganglion with high‐dose gradients outside of the target structure. The implementation of IGRT for this target had to accommodate previously placed cervical spine fusion titanium instrumentation. This hardware is rigid but beyond the treatment site. The additional use of posterior immobilization via a moldable cushion helped provide additional immobilization such that it was determined that the levels of the hardware could be used for image guidance relative to the level of the isocenter. The validity of this was evaluated and confirmed during image guidance. This allowed us to expand the useful anatomy for patient setup IGRT deviating from the generic conclusions of the phantom study as shown in Fig. 2. As far as the typicality of hardware for patients who could be considered for this treatment, these patients will have likely attempted medical management until this was deemed ineffective, then they would consider nerve‐preserving invasive procedures, followed by, with necessity, neurodestructive procedures before they could be referred to SRS. Therefore, the inherent pathway to SRS would likely result in a surgery with a spinal fusion. It is the unique nature of this pathway that has further contributed to the lack of exploration for this treatment site and added novelty to this case study. This study demonstrated that meaningful and accurate IGRT could be performed at the C2 dorsal root ganglion, even with titanium hardware near the vicinity.

Post‐treatment delivery analysis, using the results of image guidance determined for each delivered arc, showed minimal deviations from treatment planning projections. The exception to this was the maximum spinal cord dose which was projected to have received a maximum dose of 3.59 ± 0.08 Gy versus the planning calculation dose of 3.36 Gy. This deviation was noted to be well within the acceptable limits asserted by the radiation oncologist.

After treatment of trigeminal neuralgia, SRS is thought to cause axonal degeneration and necrosis, with pain relief after radiosurgery occurring from 2 to 6 weeks after the procedure.^18^ A review of three separate case series with validated pain endpoints found that the 1 year pain‐free outcome was 69% of patients, dropping to 52% at 3 year.^19^ We would propose that larger series of occipital neuralgia patients would likely mirror these results if in fact occipital neuralgia has the same underlying mechanism as trigeminal neuralgia. We are prospectively following our occipital neuralgia patients and anticipate reporting their outcomes once the sample size is statistically more valid.

Careful image registration for the actual patient was paramount for successful target definition and localization. Registration for anatomy in the cervical spine can be difficult due to the mobilization of the neck and a lack of an ability to share immobilization between diagnostic and therapeutic applications.^65^, ^66^ In addition, limitations of the rigid registration available for use with respect to the target location were noted with the unavailability of a nonrigid registration platform for this study. Such registration must be carried out with a focus on the level of C2 and with careful evaluation of the registration success for both the target‐sided dorsal root ganglion and the spinal cord and canal.

## 5 CONCLUSIONS

We report the first application of SRS for the treatment of occipital neuralgia. We performed quality assurance testing on the linear accelerator isocentricity, the fusion of planning datasets, and image guidance to ensure accurate delivery. Initial short‐term follow‐up is encouraging. Additional prospective study is needed before SRS can be considered an appropriate clinical option for occipital neuralgia.

## ACKNOWLEDGMENTS

We acknowledge Norton Healthcare for their continued support as well as the Associates in Medical Physics, LLC.
